# Mapping of multifocal breast cancer to achieve negative margins: A new step in the evolution of conservative breast surgery(A cohort study)

**DOI:** 10.1016/j.amsu.2020.05.030

**Published:** 2020-06-04

**Authors:** Yasser El Ghamrini, Tamer M.S. Salama, Mohamed I. Hassan, Haytham Mohamed Nasser

**Affiliations:** a Associate Professor of General Surgery, Ain Shams University, Cairo, Egypt; b Lecturer of Interventional Radiology, Ain Shams University, Cairo, Egypt

**Keywords:** Multifocal breast cancer, Conservative breast surgery, Negative margins, Preoperative mapping, Ultrasound-guided wiring

## Abstract

• Conservative breast surgery is the standard technique in breast cancer.
• Multifocal breast cancer is a risk factor for involved margins.
• Positive margins are considered one of the predictors for local recurrence.
• Preoperative wire mapping after breast marking by the surgeon increase the chance to have negative margins.

## 1 Introduction

Historically, mastectomy was the only type of surgery for the treatment of breast cancer, until the Milan trial in 1970. This trial introduced the concept of conservative breast surgery, for which many studies found a disease-free survival rate equivalent to that of mastectomy [1].

It is currently acknowledged that conservative breast surgery is the standard technique in early breast cancer and is broadly used in ductal carcinoma in situ (DCIS) and locally advanced breast cancer after neoadjuvant chemotherapy [2]. However, one of the main principles of conservative surgery is to achieve negative margins, as residual malignant tissue is associated with a higher rate of local recurrence (0.6%–1.5% per year) [3].

Negative margins are defined according to the Society of Surgical Oncology-American Society for Radiation Oncology (SSO-ASTRO) as “no ink on tumour” for invasive cancer, while 2 mm is enough in DCIS [4,5].

A positive margin is considered to be one of the main prognostic indicators of local recurrence in breast cancer surgery, with various factors related to the surgeon or to the tumour itself contributing to this [6]. However, reexcision or even mastectomy is the only way to cure such cases.

Multifocality is defined as the presence of two or more tumours in the same quadrant at a distance of <2–5 cm from each other. It is considered to be one of the risk factors for residual malignant cells [7].

With recent advancements in breast imaging, preoperative localization of the tumour and intraoperative frozen sections enable more accurate surgical excision and the achievement of negative margins.

Wire localization of breast cancer is a standard technique used in clinically impalpable breast cancer. However, there are no reported data on the use of the wiring technique for delineating a map for the surgeon in the case of multiple malignant foci, which would facilitate segmentectomy or quadrantectomy with a high prediction of accurate negative margins.

In this prospective study, we analyse our results for preoperative mapping of multifocal breast cancer by combining the surgeon's marking and ultrasound-guided hooked wires 1 cm from each focus and its impact on margin status. Furthermore, we suggest a new name for the technique.

## 2 Patients and methods

### 2.1 Patients’ selection

This prospective cohort study was conducted on 26 female patients who presented to our tertiary referral breast unit (Ain Shams University Hospitals) with nonmetastatic breast cancer from June 2017 to July 2019; we followed up our patients for 1–24 months. Data were collected from July 2019 to October 2019. Our study is a single-arm group with the same procedures done for all patients. All patients signed an informed consent to participate in this study, which was approved by the ethical committee of Ain Shams University held in April 2017 (IRB:0006379) with Research Registry UIN (5320). Our work has been reported in line with the STROCSS criteria [8]. We selected patients with multifocal tumours and candidates for conservative surgery with a full explanation of the outcome measures. Patients with metastatic breast cancer, locally advanced breast cancer, inflammatory breast cancer, post-neoadjuvant chemotherapy, and multicentric tumours were excluded. There were no available data in previous literature for sample size calculation. We obtained data from all patients regarding age, family history, menstrual history, size of the largest focus, location of the foci, margin status, least margin width, TNM staging, and molecular subtype.

### 2.2 Surgical procedure

All patients participating in the study were evaluated by the multidisciplinary team in our breast unit, which includes breast surgeons, a clinical oncologist, a radiologist, and a pathologist. All surgeries were therapeutic and performed by the same surgical team.

All cases underwent accurate clinical examination, bilateral sonomammography, and routine magnetic resonance imaging (MRI) with suspicions of multifocality.

### 2.3 Wire mapping

Localization of the patients' affected breast segment or quadrant was achieved under sonographic guidance, with the aim of introducing the needle to the previously planned points that surround the involved area with a safety margin of at least 1 cm. The tip of the needle was placed just about 1 cm deeper than the deep edge of the adjacent focus. This was achieved by taking the shortest pathway and as perpendicularly as possible. After confirming the needle site, we started to push the wire through the needle and then removed the needle gently, making sure not to change the wire position. After removing the needle, the wire position was confirmed via ultrasound. The procedure was then repeated at each planned point separately. In some patients, especially those with DCIS, positive identification of the lesion was very difficult and there was a cognitive fusion with other imaging modalities. MRI and mammogram as well as confirmatory biopsy were performed before planning the wire mapping. Ultrasound-guided wires were applied at the angles marked on the patient's breast, encircling all malignant foci. The wire procedure is done in multifocal breast lesions with the aim of helping the surgeon to identify the involved area (quadrant) with a good safety margin. In our study, the procedure was performed by the intervention radiologist using the Logiq P5 ultrasound machine with a 7.5–10 MHz probe. After accurate localization of the lesions by ultrasound, a virtual quadrant was drawn on the patient's skin, encircling the involved area with adequate safety margins (at least 1 cm in all directions), and the site of the wire insertion was planned. This was followed by adequate skin sterilization and local infiltrative anaesthesia at each point of insertion. A dedicated wire localizer was used (GEOTEC breast localization needle, 20 G × 7 cm). The procedure was performed using real-time sonography.

All wire mapping was performed 1 day before surgery and with the attendance of both the surgeon and the radiologist to ensure accurate localization with the chosen surgical technique and preoperative breast marking before wiring.

### 2.4 Surgical excision

In all cases, a skin incision was done according to the planned technique, and then dissection continued in the plane between the breast parenchyma and subcutaneous fat over the affected quadrant until all wires were identified. The wires were then dissected from the overlying skin so that we could plainly see the affected segment or quadrant, which was surrounded and mapped with 3–5 wires. We encircled the whole wires by marking a line using the cutting button of the cautery and kept the entire resection of the specimen with the wires in place. Radiographic images of the resected gland were then taken to document that the wires were in place, and clipping of the tumour bed was performed to facilitate the booster dose of radiotherapy.

Intraoperative frozen sections with touch preparation were used as an accurate method for margin assessment, followed by paraffin stain for all specimens.

The glandular approximation was done after dual-plane dissection to fill the cavity. A drain was inserted, followed by the closure of the skin in layers.

In nine cases, oncoplastic techniques were used to displace the excised gland (5 had inferior pedicle, 2 had vertical mammoplasty, 2 had round block).

Sentinel lymph node biopsy (SLNB) was done in twenty patients with clinically and radiologically node-negative axilla and two of them had axillary clearance.

All patients received intravenous antibiotics and analgesics, and all cases were followed up in the clinic with regular dressing. Drains were removed after 8–14 days.

All patients had regular follow-up every 3 months with annual bilateral sonomammography and breast MRI.

### 2.5 Statistical analysis

Data were collected, revised, coded, and entered into the Statistical Package for Social Sciences (IBM SPSS) version 23. The quantitative data were presented as means, standard deviations, and ranges when they are parametric and as median with interquartile range when they are nonparametric. In addition, qualitative variables were presented as numbers and percentages. The comparison between groups regarding qualitative data was done using the chi-square test. The confidence interval was set to 95%, and the margin of error accepted was set to 5%. Thus, the *P* value was considered significant at <0.05.

## 3 Results

All demographic data and tumour characteristics are shown in Table 1. The correlation of the margin status with age, lymph nodes, number of foci, size of largest focus, and molecular subtype is presented in Table 2.

**Table 1:** Patients demographic data and tumor characteristics in 26 patients with multifocal breast cancer.

Age (years)		Total no. = 26
	Mean ± SD	50.46 ± 8.86
	Range	36–65
Family history	Negative	20 (76.92%)
	Positive	6 (23.08%)
Menstrual history	Postmenopausal	10 (38.46%)
	Pre-menopausal	16 (61.54%)
Site	Right lower inner quadrant	4 (15.3%)
	Right upper outer quadrant	14 (53.8%)
	Left lower outer quadrant	4 (15.3%)
	Left upper inner quadrant	2 (7.69%)
	Right Lower outer, left lower inner	2 (7.69%)
Multifocality	Positive	26 (100.0%)
Number	Mean ± SD	2.31 ± 0.63
	Range	2–4
Size of largest focus (cm)	Mean ± SD	2.70 ± 0.69
	Range	1–3.5
Local recurrence	Positive	1 (3.8%)
Lymph vascular invasion	Negative	20 (76.9%)
	Positive	6 (23.1%)
ER	Negative	9 (34.6%)
	Positive	18 (69.2%)
PR	Negative	9 (34.6%)
	Positive	18 (69.2%)
HER-2NEU	Equivocal Positive Negative	2 (7.6%) 5 (18.5%) 19 (73.0%)
KI 67 (%)	Median (IQR)	25 (12–30)
	Range	8–60
Grade	1	2 (7.7%)
	2	18 (69.2%)
	3	6 (23.1%)
Lymph node	Median (IQR)	1 (0–2)
	Range	0–6
Margins	Negative Positive	22 (84.6%) 4 (15.3%)
Least margin (cm)	Mean ± SD	1.58 ± 0.53
	Range	0.3–2.2
Conversion to mastectomy	Positive	2 (7.6%)
Wider excision	Positive	2 (7.6%)
Pathology	Invasive duct carcinoma	15 (69.2%) 3 (11.5%) 2 (7.7) 4 (11.5%) 2 (7.7%)
	Invasive lobular carcinoma Mixed type	
	DCIS Others	
Technique	Inferior pedicle	5 (19.2%)
	V mammoplasty	2 (7.7%)
	Vertical mammoplasty Round block	2 (7.7%) 2 (7.7%)
	Standard conservative breast surgery	15 (57.7%)
Off spring	Median (IQR)	3 [[2], [3], [4], [5]]
	Range	0–7
Stage	2	20 (76.9%)
	3	6 (23.1%)
T stage	1	2 (7.7%)
	2	24 (92.3%)
N stage	0	18 (69.2%)
	1	5 (19.2%)
	2	3 (11.5%)
M stage	0	26 (100.0%)

**Table 2:** Margin status in correlation to age, number of foci, T stage, lymph node status, molecular subtype and tumor pathology.

		Negative margins	Positive margins	Test value	P-value	sig.
		No. = 22	No. = No. = 4			
Age	<50 yrs	12 (54.5%)	4 (100.0%)	2.955	0.086	NS
	≥50 yrs	10 (45.5%)	0 (0.0%)			
Number of foci	≤2	20 (90.9%)	0 (0.0%)	15.758	<0.001	HS
	>2	2 (9.1%)	4 (100.0%)			
T stage	T1	2 (9.1%)	0 (0.0%)	0.394	0.530	NS
	T2	20 (90.9%)	4 (100.0%)			
Lymph node	Negative	17 (77.27%)	2 (50.0%)	1.280	0.257	NS
	Positive	5 (22.73%)	2 (50.0%)			
Molecular subtype	Luminal A	12 (54.55%)	1 (25.0%)	0.540	0.763	NS
	Luminal B	0 (0.0%)	0 (0.0%)			
	Triple negative	8 (36.36%)	1 (25.0%)			
	HER-2	2 (9.09%)	2 (50.0%)			
Pathology	Invasive duct carcinoma	14 (63.64%)	1 (25.0%)			
	Invasive lobular carcinoma	2 (9.09%)	1 (25.0%)			
	Mixed type	2 (9.09%)	0 (0.0%)	6.027	0.197	NS
	DCIS	2 (9.09%)	2 (50.0%)			
	Others	2 (9.09%)	0 (0.0%)			

Reoperation was reported in only 2 (7.7%) patients who subsequently underwent a mastectomy, 24 (92%) passed smoothly (negative margins with no need for reoperations), and only 1 case had local recurrence after 2 years with no reported systemic recurrence.

Positive margins were reported in 4 (15.3%) cases: 2 (7.7%) were discovered in paraffin section with DCIS, and mastectomy was performed 2 weeks later, whereas the other 2 (7.7%) cases were reported during freezing (1 case with invasive ductal carcinoma and the other with invasive lobular carcinoma) with immediate reexcision until negative margins were achieved and confirmed by paraffin later.

Twenty patients had 2 malignant foci, four patients had three foci, and two patients had four lesions. The number of foci ranged from 2 to 4 (mean ± SD, 2.31 ± 0.63), which was statistically significant on the margin status (*P* = 0.001). The size of the largest focus was from 1 to 3.5 cm (2.70 ± 0.69 cm). In two patients, the size of the largest focus was <2 cm while in 24 patients the size of the largest focus was from 2.1 to 3.5 cm. All cases had T2 tumours but with no significant statistical value on the margin status. Positive nodes were found in eight patients; two patients had positive margins but with no statistically significant value in comparison to the margin status.

We followed up our patients for 1–24 months. One patient had local recurrence with triple-negative tumour and 6 positive nodes. She underwent a skin-sparing mastectomy with latissimus dorsi flap reconstruction, with the least margin width of 3 mm (1.58 ± 0.53 mm).

Three cases underwent a mastectomy, one for local recurrence after 22 months and two patients because of positive margins with DCIS.

We found that wire mapping for multifocal cancers showed a high success rate (85%) in 22 patients, frozen section failed in 2 cases (7.7%) with DCIS, and 2 cases failed by mapping.

## 4 Discussion

Conservative breast surgery followed by radiotherapy has long-term survival rates that are comparable to those of mastectomy; however, this is true only when negative margins are achieved [9].

Multifocal tumours are thought to be one of the predictors of local recurrence in breast cancer. However, most of the recent literature has stated that multiple tumours are independent risk factors for local recurrence, while other factors (infiltrated nodes, molecular subtype, and age) should be considered strongly.

No one can deny that, in multifocal cancer, especially with conservative breast surgery, it is technically demanding to achieve negative margins. A meta-analysis including 33 studies published by Houssami et al. demonstrated higher local failure with positive margins [10]. In 2002, Singletary et al. stated that there is no definite width reported for the margins that impact local recurrence; however, residual malignant cells in the tumour cavity may not be overwhelmed by adjuvant therapy [11].

In breast cancer surgery, accurate localization of the tumour with precise resection is crucial, and variable techniques have been described to ensure negative margins, including wire-guided localization, radio-guided occult lesion localization, carbon marking, intraoperative ultrasound-guided localization, cavity shave margins, and biopsy markers [12]. Localization of breast lesions using wires hooked into the tumour has been widely used to ensure easier and safer resection. However, this technique is routinely used for only small, impalpable lesions [13].

In our work, we described a different technique for the use of such wires in multifocal cancer to ensure a lower incidence of positive margins. We reported 2 (7.7%) cases with reoperation for positive margins. In 2016, Tardioli et al. found that no case had reexcision using optimized wire-guided localization [14].

In their study of a tailored needle excision with oncoplastic surgery, Fernando et al. reported positive margins in twenty patients (13.5%): 11 had DCIS, 7 had invasive cancer, and 2 had both [15].

Langhans reviewed 4118 cases, with a reexcision rate of 17.6% (725 patients) for positive margins, and found a lower reoperation rate after wire-guided excision, with a 3 times higher risk in DCIS [16].

A positive margin rate (20.8%) was reported by Laws et al. in a study conducted on 1165 patients [17]. Haloua et al. published data from the Netherlands network on positive margins after conservative breast surgery with a rate of 16.4% [18]. We reported 2 (7.5%) cases with intraoperative wire dislocation and one (3.5%) case with the inadvertent cutting of the wire. Tardioli et al. [14] reported two patients (10%) with wire displacement (*P* = 0.03) and no wire cutting during surgery [18,19]. A high rate of local failure was reported by early studies for conservative surgery in multifocal breast cancer. Conversely, recent literature has shown adequate local recurrence as long as margins are negative for each focus [18,19].

Hartsell et al. reported positive margins in 4 of 27 patients with multiple ipsilateral breast cancers, with one case of local recurrence [20]. A higher rate of local failure was stated by Kaplan et al. [6], who found that 56% of the patients had reoperation to attain negative margins [21]. In their study, Cho et al. found a significant reexcision rate (11/15); to consequently achieve clear margins, he stated that clear margins are noteworthy as a predictor for local control [22].

Conversely, Clough et al. found acceptable positive margins in multifocal (10/58) versus unifocal (23/217) tumours after oncoplastic surgery, which provides wider margins with acceptable cosmoses [23].

We reported three patients with postoperative complications, two cases with disruption and gapping of suture line that was managed conservatively and one with necrosis of the nipple-areola complex, and surgical debridement was done followed by regular dressing till the complete healing of the wound.

We can consider that studies with higher reexcision rates in patients with multiple tumours in the breast mandate some technical modification to remove each focus safely with clear-cut edges. We strongly recommend in such challenging cases (M/F) more than one process to achieve zero residual malignant cells in the tumour cavity. Thus, drawing a map by using hooked wires placed 1 cm away from the deep edge of all foci in the planned resected area will make it easier for the surgeon to perform an accurate resection. In addition, we prefer to recheck the accuracy of the wire-guided excised specimen using an intraoperative frozen section for the margins. The attendance of the surgeon and radiologist together during the mapping is important. We also found that the oncoplastic technique will provide safer resection with better cosmetic results.

There are two major limitations in our study that could be addressed in future research: first, the small number of the patients enrolled in the study, and second, the short follow-up duration for the patients.

Finally, we suggest a new term for Wire mApping oF multiFocal breast LEsions (WAFFLE), and we find it to be more indicative of the idea of encircling the whole foci, like the two pieces of the waffle encompassing the contents inside.

## 5 Conclusion

**Fig. 1:**
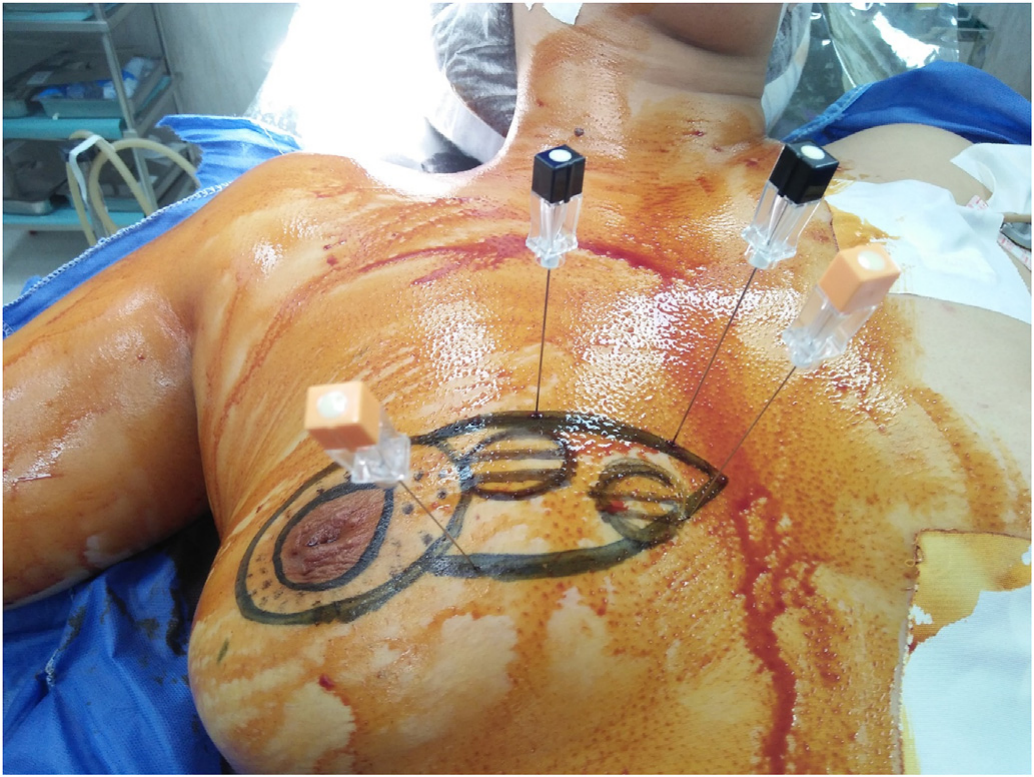
Preoperative breast marking with ultrasound guided wire mapping for bifocal breast cancer with round block technique.

**Fig. 2:**
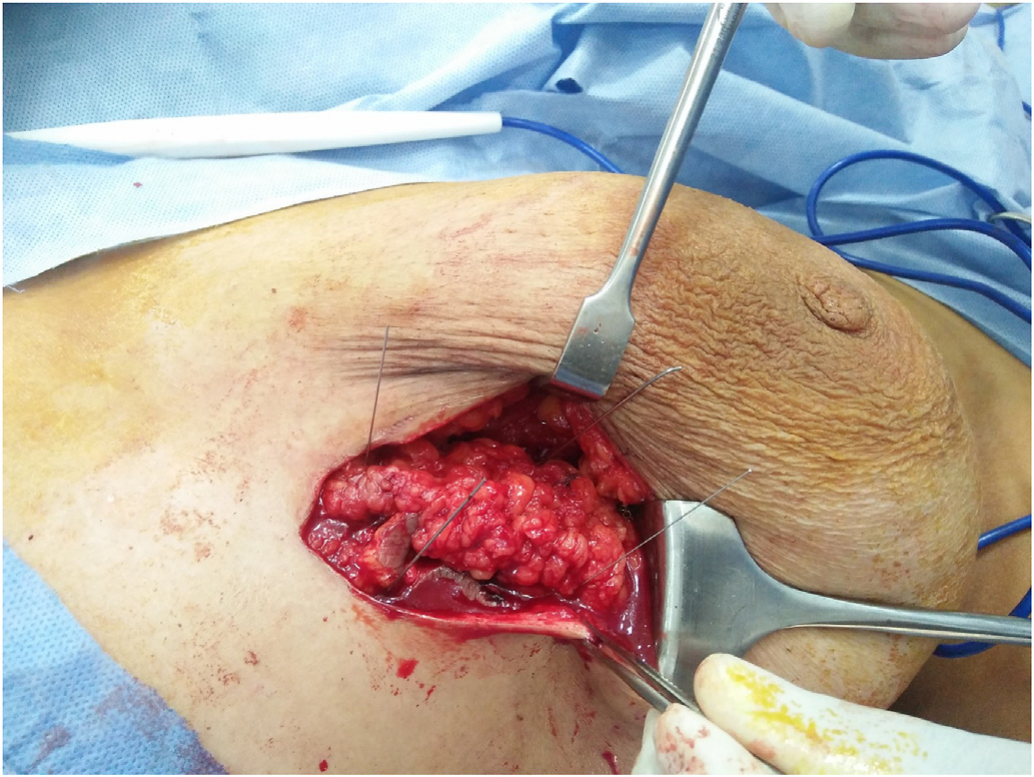
Intraoperative identification with careful dissection of the wires encircling bifocal lesions using vertical mammoplasty technique with negative margins.

In our work, we found that preoperative breast marking by the surgeon combined with mapping of the affected quadrant in multifocal breast cancer is shown to be a safe and easy technique for achieving clear margins, especially in cases with challenging multifocal lesions. A multicentre study is required to standardise this technique in surgical practice. (see Fig. 1, Fig. 2).

## Disclosures

We have no disclosures related to this manuscript.Ethical approval

The study approved in the ethical committee of general surgery department-Faculty of medicine-Ain Shams University with reference number (IRB:0006379).(see Fig. 3b, Fig. 3c, Fig. 3d, Fig. 3aA-D).

**Fig. 3a:**
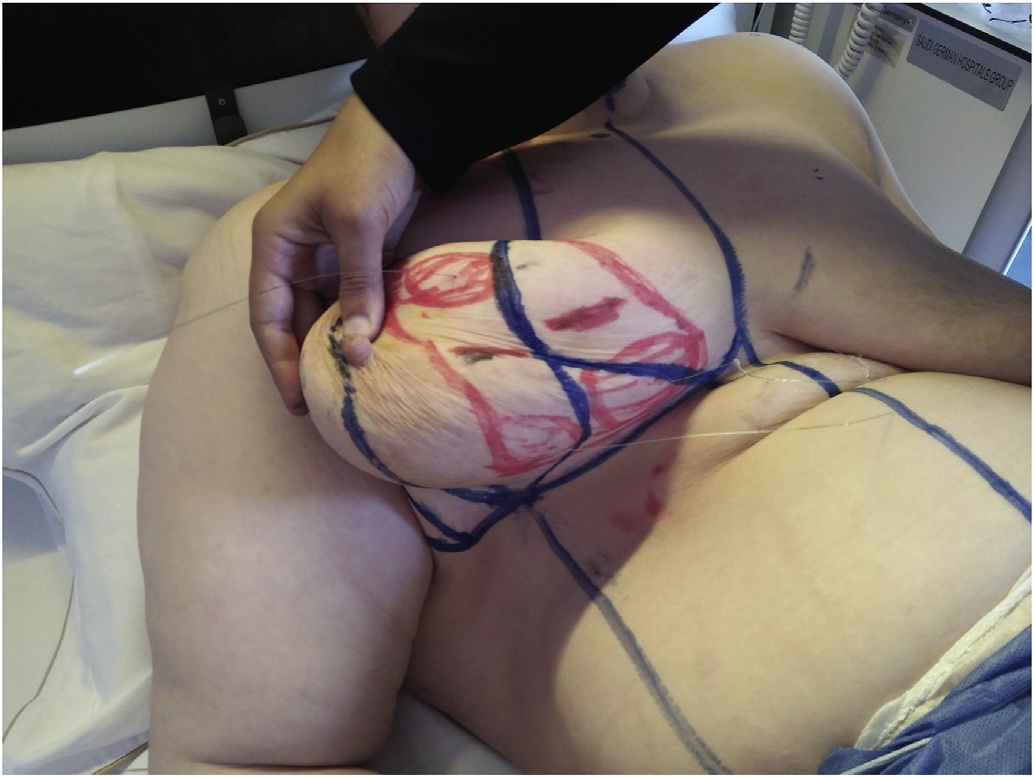
Preoperative breast marking for multifocal breast cancer with U/S guided wire mapping and inferior pedicle was done.

**Fig. 3b:**
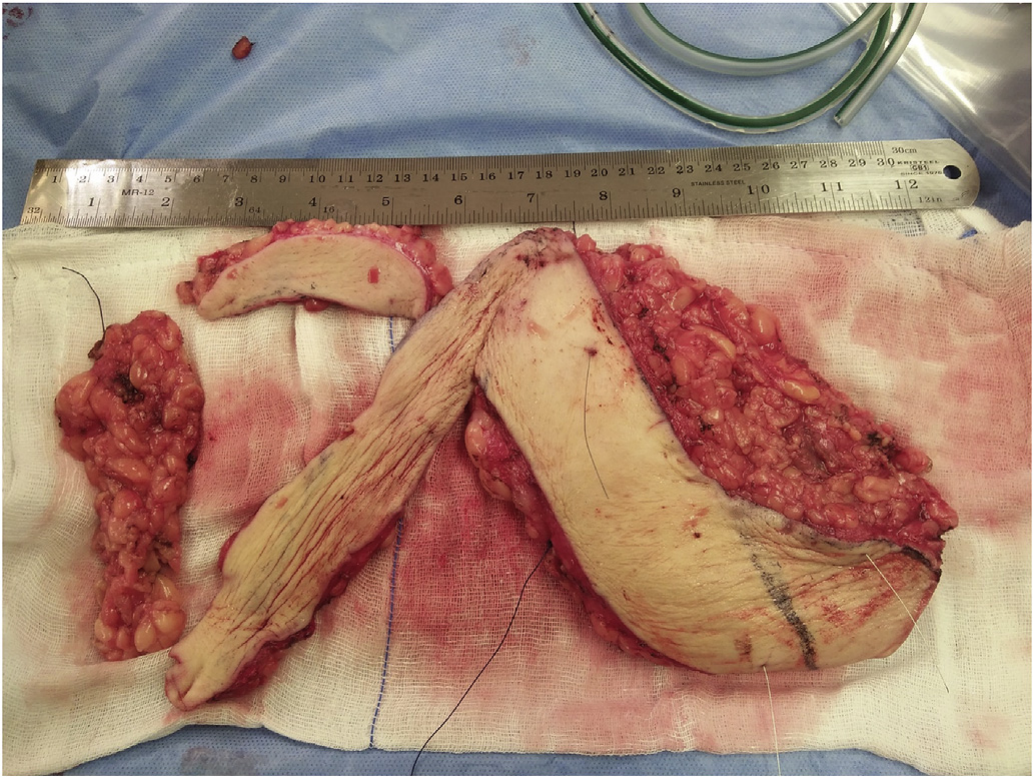
Resected specimen with wires kept in place guiding our surgical resection with negative margins.

**Fig. 3c:**
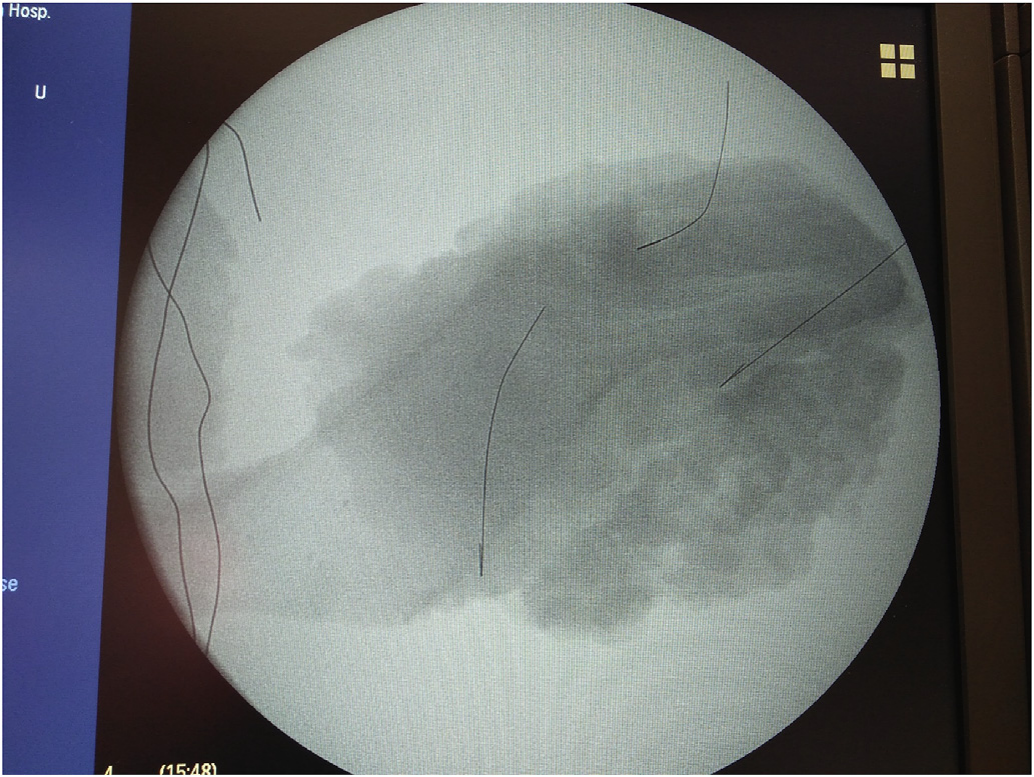
Routine imaging of the resected specimen to ensure wires kept in place.

**Fig. 3d:**
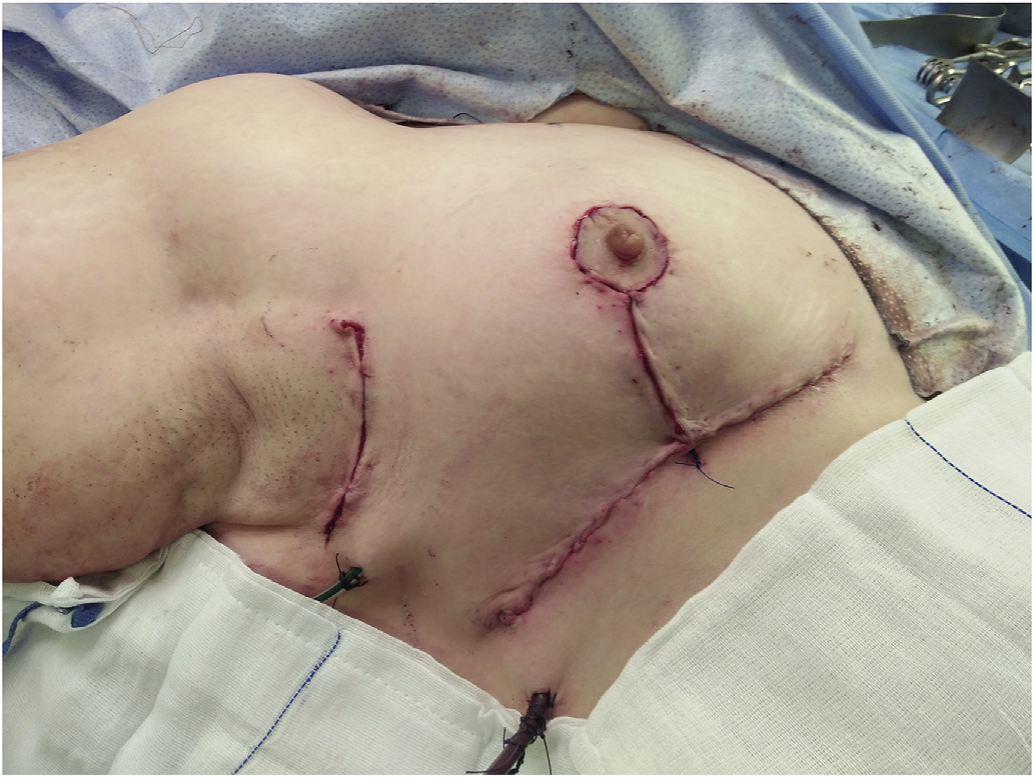
Final results after resection of multifocal breast cancer using oncoplastic technique guided by wires with negative margins.

## Author contribution

Yasser El Ghamrini: Conception and design. Acquisition, analysis and interpretation of data. Writing the article. Approved the final version.

Tamer M.S. Salama: Analysis and interpretation of data. Writing the article. Approved the final version.

Mohamed I Hassan: Analysis and interpretation of data. Writing the article. Approved the final version.

Haytham Mohamed Nasser: Analysis and interpretation of data. Writing the article. Approved the final version.

## Funding sources

There are no sources of funding.

## Registration of research studies

1 Name of the registry:Researche Registy
2 Unique Identifying number or registration ID: 5320
3 Hyperlink to the registration (must be publicly accessible):

## Guarantor

Yasser Mohamed Elghamrini

### Provenance and peer review

Not commissioned, externally peer reviewed.

## Consent

An informed written conscent was obtained from all patients sharing in the study.

## Declaration of competing interest

The authors declare that they have no conflicts of interest.
