# Supplementary material for: Mapping of multifocal breast cancer to achieve negative margins: A new step in the evolution of conservative breast surgery(A cohort study)
Source: Ann Med Surg (Lond). 2020 Jun 4;56:28–33. doi: 10.1016/j.amsu.2020.05.030 (PMC7303525; doi:10.1016/j.amsu.2020.05.030)
Supplement: Multimedia component 1 [file mmc1.docx]

| The STROCSS 2019 Guideline | | |
| --- | --- | --- |
| Item no. | **Item description** | **Page** |
| TITLE | | |
| 1 | Title:  Mapping of multifocal breast cancer to achieve negative margins: A New step in the evolution of conservative breast surgery(A prospective cohort study). | (1) |
| ABSTRACT | | |
| 2a | Introduction:  -Background  Residual malignant tissue in the tumour cavity after conservative breast surgery is associated with local recurrence. Positive margins are more commonly found in multifocal lesions, so precise resection is indispensable for attaining clear edges.  - Scientific Rationale for this study  This study evaluate the feasibility and accuracy of preoperative wire mapping after breast marking by the surgeon for the affected quadrant with multifocal cancer and its impact on the margin status in conservative breast surgery. | (1) |
| 2b | Methods:  - Study design  A prospective cohort single study conducted on 26 female patients.  - Patient populations and/or groups, including control group, if applicable  26 female patients with non-metastatic multifocal breast cancer.  - Interventions (type, operators, recipients, timeframes)  Preoperative breast marking done by the surgeon then u/s guided wires applied at the angles of a triangle or rectangle marked on patient breast encircling all malignant foci 1 cm deeper to the deep edge of each focus. All wires were identified and carefully dissected intraoperativelly with cut excision 1cm away from all wires followed by frozen section to recheck the margin status.  - Outcome measures  Margin status in multifocal breast cancer, locoregional and systemic recurrence. | (1) |
| 2c | Results:   - Summary data (with statistical relevance) with qualitative descriptions, where appropriate.   Re-operation reported only in 2(7.7%) patients had mastectomy , 24(92%) passed smoothly with only one case had local recurrence after 2years with no reported systemic recurrence.  The least margin width was 3mm with mean ± SD (1.58 ± 0.53), number of foci ranged (2-4) with Mean ± SD (2.31 ± 0.63) with significant statistical value (p=0.001).  . Wire mapping in multifocal cancer showed success rate in 85% , frozen section failed in 2 cases (7.7%) with DCIS. | (1) |
| 2d | Conclusion:   - Key conclusions   Preoperative localization with u/s guided wiring of the affected quadrant in multifocal breast cancer has shown to be safer and easier to get clear margins.  - Implications to practice  Getting clear margins in challenging multifocal breast cancer.  - Direction of and need for future research  A multicentre study is required to standardise this technique. | (1) |
| INTRODUCTION | | |
| 3 | Introduction:  - Relevant background and scientific rationale  Historically, mastectomy was the only type of surgery for the treatment of breast cancer, until the Milan 1 trial in 1970. This trial introduced the concept of conservative breast surgery, for which many studies found a disease-free survival rate equivalent to that of mastectomy^(1)^.  It is currently acknowledged that conservative breast surgery is the standard technique in early breast cancer and is broadly used in ductal carcinoma in situ (DCIS) and locally advanced breast cancer after neoadjuvant chemotherapy^(2)^. However, one of the main principles of conservative surgery is to achieve negative margins, as residual malignant tissue is associated with a higher rate of local recurrence (0.6%-1.5% per year)^(3)^.  Negative margins are defined according to the Society of Surgical Oncology–American Society for Radiation Oncology (SSO-ASTRO) as “no ink on tumour” for invasive cancer, while 2 mm is enough in DCIS^(4,5)^.  A positive margin is considered to be one of the main prognostic indicators of local recurrence in breast cancer surgery, with various factors related to the surgeon or to the tumour itself contributing to this.^(6)^ However, re-excision or even mastectomy is the only way to cure such cases.  Multifocality is defined as the presence of two or more tumours in the same quadrant at a distance of <2 to 5 cm from each other. It is considered to be one of the risk factors for residual malignant cells^(7)^.  With recent advancements in breast imaging, preoperative localization of the tumour and intraoperative frozen sections enable more accurate surgical excision and the achievement of negative margins.  Wire localization of breast cancer is a standard technique used in clinically impalpable breast cancer. However, there are no reported data on the use of the wiring technique for delineating a map for the surgeon in the case of multiple malignant foci, which would facilitate segmentectomy or quadrantectomy with a high prediction of accurate negative margins.   - Aims and objectives   In this prospective study, we analyse our results for preoperative mapping of multifocal breast cancer by combining surgeon’s marking and ultrasound guided hooked wires 1 cm from each focus and its impact on margin status, Furthermore we suggest new name for the technique. | (2)  (3) |
| METHODS | | |
| 4a | 1-Registration and ethics Name of the registry: Researche Registy  2-Unique Identifying number or registration ID: 5320 | (Author information paper) |
| 4b | Ethical Approval:  All patients signed an informed consent to participate in this study, which was approved by the ethical committee held on April 2017(IRB:0006379) | (3) |
| 4c | Protocol:  **Introduction:**  It is currently acknowledged that conservative breast surgery is the standard technique in early breast cancer and is broadly used in ductal carcinoma in situ (DCIS) and locally advanced breast cancer after neoadjuvant chemotherapy^(1)^. However, one of the main principles of conservative surgery is to achieve negative margins, as residual malignant tissue is associated with a higher rate of local recurrence (0.6%-1.5% per year) ^(2)^.  Positive margins are more commonly found in multifocal lesions, so precise resection is indispensable for attaining clear edges.  **Aim of the work**:  The aim of this study is to evaluate the feasibility and accuracy of preoperative wire mapping after breast marking by the surgeon for the affected quadrant with multifocal cancer and its impact on the margin status in conservative breast surgery.  **Patients and methods:**  The study is prospective cohort single study will be conducted on 26 female patients with non-metastatic multifocal breast cancer. Preoperative breast marking will be done by the surgeon then u/s guided wires will be applied at the angles of a triangle or rectangle marked on patient breast encircling all malignant foci 1 cm deeper to the deep edge of each focus. All wires will be identified and carefully dissected intraoperativelly with cut excision 1cm away from all wires and will be followed by frozen section to recheck the margin status.  **References:**  1- Doridot V, Nos C, Aucouturier JS, Sigal-Zafrani B, Fourquet A and Clough KB. Breast-conserving therapy of breast cancer. Cancer Radiother 2004; 8: 21-8.  2-Schnitt SJ. Risk factors for local recurrence in patients with invasive breast cancer and negativesurgical margins of excision. Where arewe andwhere arewe going? Am J Clin Pathol 2003;120(4):485–8. | (1) |
| 4d | Patient Involvement in Research  - Patients involved in study were selected from breast clinic with multifocal breast cancer with full explanation of the procedure and the outcome measures. | (3) |
| 5a | Study Design:   - A single-centred prospective cohort study was done | (3) |
| 5b | Setting:  Geographical location  The study was held in Cairo-Egypt  - Nature of institution (e.g. academic/community, public/private)  All patients were diagnosed and operated in a university hospital.  - Dates (recruitment, exposure, follow-up, data collection)  Recruitment started in June2017, we operated our patients from June2017 to July2019.Patients were followed up from 1-24 months. Data were collected from July2019 to October2019. | (3) |
| 5c | Cohort Groups:  Our study is a single arm group with the same procedures done for all patients. | (3) |
| 5d | Subgroup Analysis:  -No subgroups in our study. | (3) |
| 6a | Participants:   - Eligibility criteria   We selected patients with multifocal tumours and candidates for conservative breast surgery.  - Recruitment sources  All patients were recruited from the breast clinic in Ain Shams university   - Length and methods of follow-up   Patients were followed up for 24months by clinical and radiological examination. | (3) |
| 6b | Recruitment:  - Methods of recruitment to each patient group  All patients were recruited after clinical, radiological and pathological diagnosis for non-metastatic multifocal breast cancer candidate for conservative breast surgery  - Period of recruitment  Period of recruitment was from june2017 to July2019. | (3) |
| 6c | Sample Size:  There was no available data in previous literature for sample size calculation. | (3) |
| INTERVENTION AND CONSIDERATIONS | | |
| 7a | Pre-intervention Considerations:   - Patient optimisation (pre-surgical measures)   All patients participating in the study were evaluated by the multidisciplinary team in our breast unit, which includes breast surgeons, a clinical oncologist, radiologist, and pathologist. All surgeries were performed by the same surgical team.  -All cases underwent accurate clinical examination, bilateral sonomammography, and routine magnetic resonance imaging (MRI) with suspicions of multifocality.   - Pre-intervention treatment   All patients with medical problems were assed preoperatively. | (3) |
| 7b | Intervention:   - All patients underwent conservative breast surgery for breast cancer. - All surgeries were therapeutic. - All patients received regular post-surgical medications (antibiotics, - analgesics, intravenous fluids). - Manufacturer and model details where not applicable. | (3,4,5) |
| 7c | Intra-Intervention Considerations:  In all cases, skin incision was done according to the planned technique, and then dissection continued in the plane between the breast parenchyma and subcutaneous fat over the affected quadrant until all wires were identified. The wires were then dissected from the overlying skin so that we could plainly see the affected segment or quadrant, which was surrounded and mapped with 3-5 wires. We encircled whole wires by marking a line using the cutting button of the cautery and kept the entire resection of the specimen with the wires in place. Radiographic images of the resected gland were then taken to document that the wires were in place, and clipping of the tumour bed was performed to facilitate the booster dose of radiotherapy.  Intraoperative frozen sections with touch preparation were used as an accurate method for margin assessment, followed by paraffin stain for all specimens.  Glandular approximation done after dual-plane dissection to fill the cavity. A drain was inserted, followed by closure of the skin in layers.  In 9 cases, oncoplastic techniques were used to displace the excised gland (5 had inferior pedicle, 2 vertical mammoplasty, 2 round block, and 2 V mammoplasty).  Sentinel lymph node biopsy (SLNB) was done in twenty patients with clinically and radiologically node negative axilla and two of them had axillary clearance.  All patients received intravenous antibiotics, analgesics with follow up of all cases was done in the clinic with regular dressing. Drains were removed after 8 to 14 days.    - Figures illustrated in list of figures. | (5,6) |
| 7d | Operator Details: These kind of surgeries requires special training with long learning curve for these highly specialized techniques. | (3) |
| 7e | Quality Control:   - We standardise selection criteria for patients sharing in the study with all surgical and radiological procedures were done by the same team.   All patients participating in the study were evaluated by the multidisciplinary team in our breast unit, which includes breast surgeons, a clinical oncologist, radiologist, and pathologist. All surgeries were therapeutic and performed by the same surgical team.   - All surgeries done by highly specialized well trained consultants in breast cancer and reconstructive surgery, also wiring technique done by consultant of interventional radiology.   Strict follow up for all patients | (3) |
| 7f | Post-Intervention Considerations:   - Post-operative instructions and care   Follow up of all cases was done in the clinic with regular dressing. Drains were removed after 8 to 14 days.   - Follow-up measures   All patients followed up for post-surgical complications(wound infection, wound dehiscence, nipple and areola necrosis   - Future surveillance requirements (e.g. imaging, blood tests)   patients were followed up by annual sonomammography , MRI breast and PET-CT. | (5) |
| 8 | Outcomes: the following areas are described comprehensively   - Primary outcomes, including validation, where applicable   Margins in multifocal breast cancer   - Definitions of outcomes   Negative margins in multifocal breast cancer is defined as at least 2mm free cut edges   - Secondary outcomes, where appropriate   Locoregional and systemic recurrence of multifocal breast cancer  Follow-up period for outcome assessment, divided by group   - We followed up our patients for 1 to 24 months. One patient had local recurrence with no systemic recurrence. | (7) |
| 9 | Statistics:   - Data were collected, revised, coded, and entered into the Statistical Package for Social Sciences (IBM SPSS) version 23. The quantitative data were presented as means, standard deviations, and ranges when parametric and as median with interquartile range when nonparametric. In addition, qualitative variables were presented as number and percentages. The comparison between groups regarding qualitative data was done using chi-square test. The confidence interval was set to 95%, and the margin of error accepted was set to 5%. Thus, the *p*-value was considered significant at <0.05. - Confounders and their control, if known   Confounders were controlled from the start by exclusion of patients with any criteria suspected to be a confounder in this study.   - Analysis approach (e.g. intention to treat/per protocol)   Per protocol analysis was done (all study participants completed follow up period)   - No subgroups | (6) |
| RESULTS | | |
| 10a | Participants: the following areas are described comprehensively   - Flow of participants (recruitment, non-participation, cross-over and withdrawal, with reasons)   All patients were recruited from university breast clinic with non-metastatic breast cancer and candidate for conservative breast surgery while patients with metastatic cancer, inflammatory breast cancer post neoadjuvant chemotherapy and multicentric tumours were excluded.   - Population demographics (prognostic features, relevant socioeconomic features, and significant numerical differences)   - Demographic data were collected for all patients in table(A),patients enrolled in the study had variable socioeconomic level | (3) |
| 10b | Participant Comparison: the following areas are described comprehensively  Table comparing demographics included   \|  \| \| **Total no. = 26** \| \| --- \| --- \| --- \| \| Age (years) \| Mean ± SD \| 50.46 ± 8.86 \| \| Range \| 36 – 65 \| \| Family history \| Negative \| 20 (76.92%) \| \| Positive \| 6 (23.08%) \| \| Menstrual history \| Postmenopausal \| 10 (38.46%) \| \| Pre-menopausal \| 16 (61.54%) \| \| Site \| Right lower inner quadrant \| 4 (15.3%) \| \| Right upper outer quadrant \| 14 (53.8%) \| \| Left lower outer quadrant \| 4 (15.3%) \| \| Left upper inner quadrant \| 2 (7.69%) \| \| Right Lower outer ,left lower inner \| 2 (7.69%) \| \| Multifocality \| Positive \| 26 (100.0%) \| \| Number \| Mean ± SD \| 2.31 ± 0.63 \| \| Range \| 2 – 4 \| \| Size of largest focus(cm) \| Mean ± SD \| 2.70 ± 0.69 \| \| Range \| 1 – 3.5 \| \| Local recurrence \| Positive \| 1 (3.8%) \| \| Lymph vascular invasion \| Negative \| 20 (76.9%) \| \| Positive \| 6 (23.1%) \| \| ER \| Negative \| 9 (34.6%) \| \| Positive \| 18 (69.2%) \| \| PR \| Negative \| 9 (34.6%) \| \| Positive \| 18 (69.2%) \| \| HER-2NEU \| Equivocal  Positive  Negative \| 2 (7.6%)  5 (18.5%)  19 (73.0%) \| \| KI 67 (%) \| Median (IQR) \| 25 (12 – 30) \| \| Range \| 8 – 60 \| \| Grade \| 1 \| 2 (7.7%) \| \| 2 \| 18 (69.2%) \| \| 3 \| 6 (23.1%) \| \| Lymph node \| Median (IQR) \| 1 (0 – 2) \| \| Range \| 0 – 6 \| \| Margins \| Negative  Positive \| 22 (84.6%)  4(15.3%) \| \| Least margin (cm) \| Mean ± SD \| 1.58 ± 0.53 \| \| Range \| 0.3 – 2.2 \| \| Conversion to mastectomy \| Positive \| 2 (7.6%) \| \| Wider excision \| Positive \| 2(7.6%) \| \| Pathology \| Invasive duct carcinoma \| 15 (69.2%)  3 (11.5%)  2(7.7)  4(11.5%)  2 (7.7%) \| \| Invasive lobular carcinoma  Mixed type \| \| DCIS  Others \| \| (Technique \| Inferior pedicle \| 5 (19.2%) \| \| V mammoplasty \| 2 (7.7%) \| \| Vertical mammoplasty  Round block \| 2(7.7%)  2 (7.7%) \| \| Standard conservative breast surgery \| 15 (57.7%) \| \| Off spring \| Median (IQR) \| 3 (2 – 5) \| \| Range \| 0 – 7 \| \| Stage \| 2 \| 20 (76.9%) \| \| 3 \| 6 (23.1%) \| \| T stage \| 1 \| 2 (7.7%) \| \| 2 \| 24 (92.3%) \| \| N stage \| 0 \| 18 (69.2%) \| \| 1 \| 5 (19.2%) \| \| 2 \| 3 (11.5%) \| \| M stage \| 0 \| 26 (100.0%) \|  - Differences, with statistical relevance   The number of foci ranged from 2 to 4 (mean ± SD, 2.31 ± 0.63), which was statistically significant on the margin status (*p* = 0.001).   - Any group matching, with methods   only one group, no comparison | (List of tables)    (7) |
| 10c | Intervention: the following areas are described comprehensively   - Changes to interventions, with rationale and diagram, if appropriate   Wire localization of breast cancer is a standard technique used in clinically impalpable breast cancer. However, there are no reported data on the use of the wiring technique for delineating a map for the surgeon in the case of multiple malignant foci   - Learning required for interventions - Application of this technique mandates well trained surgeon in breast cancer surgery and how to deal with multiple wires in the breast during surgery, also trained radiologist for wiring of multiple breast lesions. - Degree of novelty for intervention   This technique considered a new novel technique to have negative margins in multifocal breast cancer. | (2) |
| 11a | Outcomes: the following areas are described comprehensively   - Clinician-assessed and patient-reported outcomes for each group   We found that we succeeded to have negative margins in 85% of our patients.   - Relevant photographs and imaging are desirable - Photographs are illustrated in list of figures. - Confounders to outcomes and which are adjusted   confounders were controlled by exclusion criteria) | (7) |
| 11b | Tolerance: the following areas are described comprehensively   - Assessment of tolerance   not applicable in this study   - Loss to follow up, with reasons (percentage and fraction)   We have no patients lost in the follow up   - Cross-over with explanation   no cross over the study has only one group | (7) |
| 11c | Complications: the following areas are described comprehensively   - Adverse events described   We reported 3 patients with postoperative complications ,two cases had disruption and gapping of suture line that was managed conservatively and one with necrosis of the nipple-areola complex and surgical debridement was done followed by regular dressing till complete healing of the wound.   - The complications in our study according to Clavien-Dindo classification are grade 1. - Mitigation for adverse events (blood loss, wound care, revision surgery should be specified)   We had two patients had revision of surgery for positive margins, two others had regular wound care for wound dehiscence. | (7,9) |
| 12 | Key Results:   - Key results, including relevant raw data   We found that wire mapping for multifocal cancers showed high success rate (85%) in 22 patients, frozen section failed in 2 cases (7.7%) with DCIS and detected 2 cases failed by mapping   - Statistical analyses with significance   The number of foci ranged from 2 to 4 (mean ± SD, 2.31 ± 0.63), which was statistically significant on the margin status (*p* = 0.001). | (7) |
| DISCUSSION | | |
| 13 | Discussion:  Conservative breast surgery followed by radiotherapy has long-term survival rates that are comparable to those of mastectomy; however, this is true only when negative margins are achieved ^(8)^.  Multifocal tumours are thought to be one of the predictors of local recurrence in breast cancer. However, most of the recent literature has stated that multiple tumours are independent risk factors for local recurrence, while other factor (infiltrated nodes, molecular subtype, and age) should be considered strongly.  No one can deny that in multifocal cancer, especially with conservative breast surgery, it is technically demanding to achieve negative margins. A meta-analysis including 33 studies published by Houssami and colleagues demonstrated higher local failure with positive margins ^(9)^. In 2002, Singletary et al. stated that there is no definite width reported for the margins that impacts local recurrence; however, residual malignant cells in the tumour cavity may not be overwhelmed by adjuvant therapy ^(10)^.  In breast cancer surgery, accurate localization of the tumour with precise resection is crucial, and variable techniques have been described to ensure negative margins, including wire-guided localization, radio-guided occult lesion localization, carbon marking, intraoperative ultrasound-guided localization, cavity shave margins, and biopsy markers ^(11)^. Localization of breast lesions using wires hooked into the tumour has been widely used to ensure easier and safer resection. However, this technique is routinely used for only small, impalpable lesions^(12)^.  In our work, we described different technique for the use of such wires in multifocal cancer to ensure a lower incidence of positive margins. We reported 2 (7.7%) cases with reoperation for positive margins. In 2016, Tardioli et al. found that no case had re-excision using optimized wire guided localization^(13)^.  In their study of a tailored needle excision with oncoplastic surgery, Fernando et al reported positive margins in 20 patients (13.5%): 11 had DCIS, 7 had invasive cancer, and 2 had both^(14)^.  Langhans reviewed 4118 cases, with a re-excision rate of 17.6% (725 patients) for positive margins and found a lower reoperation rate after wire-guided excision, with a 3 times higher risk in DCIS^(15)^.  A positive margin rate (20.8%) was reported by Laws et al. in a study conducted on 1165 patients. (16) Haloua et al. published data from the Netherlands network on positive margins after conservative breast surgery with a rate 16.4%^(17)^. We reported 2 (7.5%) cases with intraoperative wire dislocation and one (3.5%) case with inadvertent cutting of the wire. Tardioli et al ^(13)^. reported two patients with wire displacement 10% (*p* = 0.03) and no wire cutting during surgery^(18,19)^. A high rate of local failure was reported by early studies for conservative surgery in multifocal breast cancer. Conversely, recent literature has shown adequate local recurrence as long as margins are negative for each focus ^(18,19)^.  Hartsell et al. reported positive margins in 4 of 27 patients with multiple ipsilateral breast cancer, with 1 case of local recurrence ^(20)^. A higher rate of local failure was stated by Kaplan et al.^(6)^, who found 56% of the patients had reoperation to attain negative margins^(21)^. In their study, Cho et al. found a significant re-excision rate (11/15); to consequently achieve clear margins, he stated that clear margins are noteworthy as a predictor for local control ^(22)^.  Conversely, Clough found acceptable positive margins in multifocal (10/58) versus unifocal (23/217) after oncoplastic surgery, which provides wider margins with acceptable cosmoses ^(23)^.  We reported 3 patients with postoperative complications ,two cases had disruption and gapping of suture line that was managed conservatively and one with necrosis of the nipple-areola complex and surgical debridement was done followed by regular dressing till complete healing of the wound.  We can consider that studies with higher re-excision rates in patients with multiple tumours in the breast mandates some technical modification to remove each focus safely with clear-cut edges. We strongly recommend in such challenging cases (M/F) more than one process to achieve zero residual malignant cells in the tumour cavity. Thus, drawing a map by using hooked wires placed 1 cm away from the deep edge of all foci in the planned resected area will make it easier for the surgeon to perform an accurate resection. In addition, we prefer to recheck the accuracy of the wire-guided excised specimen using intraoperative frozen section for the margins. The attendance of the surgeon and radiologist together during  the mapping is key. We also found that the oncoplastic technique will provide safer resection with better cosmetic results.  There are two major limitations in our study that could be addressed in future research. First, the small number of the patients enrolled in the study second the short follow up duration for the patients.  Finally, we suggest a new term for wire mapping of multifocal breast lesions (WAFFLE), and we find it to be more indicative of the idea of encircling the whole foci, like the two pieces of the waffle encompassing the contents inside. | (8,9.  10) |
| 14 | Strengths and Limitations:   - Strengths of the study   Our study deals with a new technique for a challenging problem in breast cancer surgery.  All surgeons, radiologists sharing in the study are senior consultants and highly expert in this field.  -All cases were discussed in our university multidesplinary team.     - Limitations and potential impact on results   There are two major limitations in our study that could be addressed in future research. First, the small number of the patients enrolled in the study second the short follow up duration for the patients.   - Assessment of bias and management   we tried to avoid sources of bias by exclusion of patients with metastatic, inflammatory breast cancer and multi-centeric breast cancer | (3,10) |
| 15 | Implications and Relevance: the following areas are described comprehensively   - Relevance of findings and potential implications to clinical practice are detailed   Our findings showed high success rate in 85% of patient with great implication on the margin status of multifocal breast cancer.   - Future research that is needed is described, with study designs detailed - Future and multicentre study is needed with larger numbers and longer follow up to standardize this technique. | (7,10) |
| CONCLUSION | | |
| 16 | Conclusions:   - In our work, we found that preoperative breast marking by the surgeon combined with mapping of the affected quadrant in multifocal breast cancer is shown to be a safe and easy technique for achieving clear margins, especially in cases with challenging multifocal lesions.     - A multicentre study is required to standardise this technique in the surgical practice. | (10) |
| DECLARATIONS | | |
| 17a | Conflicts of interest  -No conflicts of interest. | (10) |
| 17b | Funding   - No sources of funding | (10) |
